# Tailored Functionalized Protein Nanocarriers for Cancer Therapy: Recent Developments and Prospects

**DOI:** 10.3390/pharmaceutics15010168

**Published:** 2023-01-03

**Authors:** Mohamed A. A. Abdelhamid, Mi-Ran Ki, Amer Ali Abd El-Hafeez, Ryeo Gang Son, Seung Pil Pack

**Affiliations:** 1 Department of Biotechnology and Bioinformatics, Korea University, Sejong-Ro 2511, Sejong 30019, Republic of Korea; 2 Department of Botany and Microbiology, Faculty of Science, Minia University, Minia 61519, Egypt; 3 Institute of Industrial Technology, Korea University, Sejong-Ro 2511, Sejong 30019, Republic of Korea; 4 Pharmacology and Experimental Oncology Unit, Cancer Biology Department, National Cancer Institute, Cairo University, Cairo 11796, Egypt

**Keywords:** cancer therapy, drug delivery, protein nanoparticles, controlled release, targeting, self-assembly, nanocage

## Abstract

Recently, the potential use of nanoparticles for the targeted delivery of therapeutic and diagnostic agents has garnered increased interest. Several nanoparticle drug delivery systems have been developed for cancer treatment. Typically, protein-based nanocarriers offer several advantages, including biodegradability and biocompatibility. Using genetic engineering or chemical conjugation approaches, well-known naturally occurring protein nanoparticles can be further prepared, engineered, and functionalized in their self-assembly to meet the demands of clinical production efficiency. Accordingly, promising protein nanoparticles have been developed with outstanding tumor-targeting capabilities, ultimately overcoming multidrug resistance issues, in vivo delivery barriers, and mimicking the tumor microenvironment. Bioinspired by natural nanoparticles, advanced computational techniques have been harnessed for the programmable design of highly homogenous protein nanoparticles, which could open new routes for the rational design of vaccines and drug formulations. The current review aims to present several significant advancements made in protein nanoparticle technology, and their use in cancer therapy. Additionally, tailored construction methods and therapeutic applications of engineered protein-based nanoparticles are discussed.

## 1. Introduction

Over the past few decades, biomedical research has increasingly focused on cancer treatment owing to its profound threat to human health [1,2,3]. As the mortality rate of cancer increases annually, more effective cancer therapeutic strategies are needed [4,5]. Notably, antitumor drugs cannot be delivered selectively to the target tissue, thereby prompting the need for effective drug delivery systems [6,7]. Nanotechnology is expected to fundamentally change the approaches taken by pharmaceutical and biotechnology industries in the near future [8]. Many novel carriers have been developed through nanotechnology and these carriers provide controlled release and targeted delivery of a range of therapeutic molecules, including proteins, peptides, genes, chemical drugs, and growth factors [9,10].

Interestingly, nanoparticles can be exploited to improve drug delivery, such as drug site-specific targeting, bioavailability, and biodistribution, in addition to preventing drug degradation, increasing circulation times, and reducing cytotoxicity against normal cells. Current nanoscale drug delivery systems include metallic nanoparticles, liposomes, and protein nanoparticles [11]. Nanoparticles are designed as a delivery system to achieve site-specific effects by controlling the particle size, surface properties, and release rates of pharmacologically active agents [12]. Different nanoparticles have been investigated to improve the therapeutic index, including natural, chemically synthesized, recombinant, and hybrid materials [13,14,15]. Additionally, uniform and precise functionalization of multiple elements is required to design successful drug delivery using these nanoparticles.

Although remarkable progress has been made in the development of natural and chemically derived nanoparticles, genetically encoded nanoparticle synthesis offers unique opportunities for nearly complete control over the stereochemistry, structure, and self-assembly behavior of nanoparticles. Additionally, genetic engineering approaches produce recombinant nanoparticles with controlled properties, such as surface charge, environmental responsiveness, drug encapsulation, stability, and ligand display to the extent that either natural or synthetically derived nanoparticles cannot replicate. In this review, we aimed to summarize the status of naturally occurring and de novo designs of protein nanoparticles that self-assemble into nanoscale biomaterials and their tailored uses to deliver therapeutic drugs effectively. In addition, we discussed how engineered nanoparticle biomaterials can be constructed, and highlighted their physicochemical properties, self-assembly into nanoparticles, tailored functionalization, and pharmacological properties.

## 2. Engineered Design of Naturally Occurring Self-Assembled Protein Nanoparticles

With advancements in recombinant technology, an entirely distinct platform is now available for engineering proteins with different functionalities and properties to meet the needs of various delivery approaches for cancer nanotherapeutics. Several microorganisms and cell lines have been used as biofactories to produce fusion protein nanoparticles that reduce immunological responses and save time in a cost-effective manner. Several geometries commonly associated with naturally occurring self-assembled proteins can lead to distinct three-dimensional particle-like structures. These assembled proteins often serve as flexible therapeutic delivery platforms owing to their unique architectures. Several of these protein cages and polymers, such as recombinant ferritins (Fn), vaults, encapsulins, small heat shock proteins, and elastin-like polypeptides, are the most studied self-assembled protein nanostructures for therapeutic delivery. The recombinant production and functionalization of these protein nanoparticles, in addition to the developed methods for cargo loading and representative applications, are highlighted in Table 1. The unique and functionalized structures of these nanoparticles can provide excellent tumor-targeting properties, biocompatibility, and favorable pharmacokinetics, thereby facilitating the targeting of diagnostic molecules and therapeutic drugs to specific targets (Table 1).

### 2.1. Application of Engineered Fn Nanoparticles in Therapeutics Delivery

One of the commonly used nanocages for drug delivery is Fn [16,17]. Fn nanoparticles are oligomeric proteins composed of self-assembled 24-subunits, with exterior and interior diameters of 12 and 8 nm, respectively [16]. Drug molecules can be loaded and encapsulated into the inner core of Fn. Fn is highly biocompatible, biodegradable, and has low toxicity, which are desirable properties of nanocarriers [18]. Owing to its sensitivity to pH, the nanocage-like structure of Fn facilitates various drug-loading techniques (Table 1) [19]. For instance, Fn disassembles in strongly acidic environments but reassembles once the pH levels return to physiological levels. Therefore, therapeutic drugs can be encapsulated within Fn via the manipulation of their disassembly and reassembly. Accordingly, several drugs have been loaded and delivered by harnessing Fn’s unique properties.

**Table 1:** Genetic preparation of natural-occurring protein nanoparticles and their functionalization for cancer therapy applications.

Protein Name	Protein Source	Expression System	Loading Cargo	Loading Method	Application	Reference
Ferritins	Human heavy chain	*E. coli*	Paclitaxel ^a^	pH ^a^	Targeted drug delivery to glioma cancer cells	[20]
	Human heavy chain	*E. coli* BL21(DE3)	Doxorubicin ^a^	pH ^a^	Efficient internalization of TfR1-positive cancer cells	[21]
	Human heavy chain	*E. coli* BL21(DE3)	Doxorubicin ^a^	One-step incubation ^a^	Superior drug loading capacity and encapsulation efficiency	[22]
	Human heavy chain	*E. coli* Rosetta(DE3)	CpG ODNs ^a^/M2pep ^b^	pH ^a^/genetic engineering ^b^	Target delivery of oligodeoxynucleotides to M2-type TAMs	[23]
	Human heavy chain	*E. coli* BL21(DE3)	Luciferin ^b^	Chemical conjugation ^b^	Tracking cancer nanodrug delivery through bioluminescence	[24]
	Human heavy chain	*E. coli*	Cisplatin ^a^/antibody Ep1 ^b^	pH ^a^/chemical conjugation ^b^	Site-specific targeting melanoma cells	[25]
	*Helicobacter pylori*	*E. coli* BL21(DE3)	Doxorubicin ^a^	pH ^a^	Controlled release of the drug	[26]
	*Helicobacter pylori*	*E. coli* BL21(DE3)	Doxorubicin ^a^/paclitaxel ^b^	pH ^a^/self-entrapment in artificial shell ^b^	Dual drug delivery	[27]
Vaults	Rat major vault protein (MVP)	Sf9 cells (insects)	Lytic pVI peptide ^b^	Genetic engineering ^b^	Enhance the nanoparticle’s escape from the endosomal compartment	[28]
	Rat MVP and MVP interaction domain	Sf9 cells	Lymphoid chemokine ^a^	Genetic engineering ^a^	Target delivery with growth inhibition of lung cancer cells	[29]
	Recombinant human vault	Sf9 cells	Antiretroviral drugs ^b^	Chemical conjugation ^b^	Inhibition of HIV-1 infection	[30]
	Rat MVP	Sf9 cells	Epidermal growth factor ^b^	Genetic engineering ^b^	Enhancing the nanoparticles binding to epithelial cancer cells (A431)	[31]
	Rat MVP and MVP interaction domain	Human cell line	Enhanced green fluorescence protein ^a^	Genetic engineering ^a^	Efficient production of recombinant vaults loaded cargos for drug delivery	[32]
Encapsulins	*Thermotoga maritima*	*E. coli* BL21(DE3)	Cell binding peptide(SP94) ^b^	Genetic engineering ^b^	Selective targeted drug delivery to HepG2 cells	[33]
	*T. maritima*	*E. coli* BL21(DE3)	Cell binding peptide (SP94) ^b^/aldoxorubicin ^b^	Chemical conjugation ^b^	Selective targeted drug delivery to HepG2 cells	[33]
	*T. maritima*	*E. coli* BL21(DE3)	Fc-binding peptide ^b^	Genetic engineering ^b^	Selective targeting to SCC-7 cell line	[34]
	*T. maritima*	*E. coli* BL21Star(DE3)	miniSOG ^a^/designed Ankyrin repeat protein ^b^	Self-encapsulation ^a^/genetic engineering ^b^	Selective targeting to HER2 positive breast cancer cells and triggering apoptosis	[35]
	*T. maritima*	*E. coli* Rosetta	Split-C-intein ^a^/SpyTag ^b^	Genetic engineering ^a,b^	An efficient approach for multifunctional therapeutic loading	[36]
Small heat shock proteins	*Methanococcus jannaschii*	*E. coli*	Doxorubicin ^a^	Chemical conjugation ^a^	Controlled drug release	[37]
	*M. jannaschii*	*E. coli* BL21(DE3)	RGD-4C ^b^	Genetic engineering ^b^	Cell-specific targeting	[38]
	*M. jannaschii*	*E. coli*	SP94 ^b^	Genetic engineering ^b^	Selective targeting to HepG2 cells	[39]
	*M. jannaschii*	Recombinantly expressed	Paclitaxel ^a^/TAT peptide ^b^	Encapsulation ^a^/chemical conjugation ^b^	Development of tumor microenvironment-targeting nanoparticles	[40]
Elastin-like polypeptides	Engineered polypeptides inspired by natural tropoelastin	*E. coli* BL21(DE3)	Antitumor peptide ^b^	Genetic engineering ^b^	Selective delivery system to colon adenocarcinomas	[41]
		*E. coli* BLR(DE3)	Paclitaxel ^a^/ iRGD ^b^	Encapsulation ^a^/genetic engineering ^b^	Development of active targeting and cell-penetrating nanoparticles	[42]
		*E. coli* BLR(DE3)	Doxorubicin ^a^/cell penetrating peptide ^b^	Chemical conjugation ^a^/genetic engineering ^b^	Specific drug delivery	[43]
		*E. coli* SHuffle T7	Anti-programmed death-1 (αPD-1) scFv ^b^	Genetic engineering ^b^	Blocking the PD-1 immune checkpoint	[44]
		ClearColi BL21(DE3)	Anti-CD99 scFv ^b^	Genetic engineering ^b^	Development of multivalent scFV nanoworms with strong anti-leukemic effects	[45]

^a^ Interior space of protein nanoparticles. ^b^ Exterior surface of protein nanoparticles.

Lui et al. constructed human ferritin (HFn) loaded with paclitaxel (PTX) as an antitumor nanocage for the treatment of glioma [20]. This HFn-PTX selectively binds to transferrin receptor 1 (TFR1) in glioma cells to enhance PTX delivery. With a median survival of 30 days, HFn-PTX exhibited a superior antitumor effect compared to physiological saline (13 days) and free PTX (14 days). In another study, doxorubicin (DOX)-loaded HFn, which served as a “Trojan Horse”, internalized cancer cells more quickly and efficiently than free DOX, and was then promptly relocated to the nucleus when the partial release of DOX in the cytoplasm caused DNA damage [21].

Although Fn nanoparticles represent innovative platforms for therapeutic nanocarriers, the current technique for drug encapsulation requires further advancement, which may restrict its clinical use. One major challenge is the construction of a drug-Fn complex with a high loading capacity inside the nanocage; this is because there is no mechanism for drug accumulation. As a result, Inoue et al. created a one-step approach for high anticancer drug loading in Fn nanoparticles [22]. The DOX/Fn complex was obtained by incubating the drug and Fn nanoparticles in buffer at pH 9. A small Fn cavity and electric charge potential facilitate the movement of drug molecules through the pores, enabling their deposition in the Fn cavity. Compared to the low loading capacity of 3–4% for the disassembly/assembly approach, the established one-step process achieved a loading capacity and encapsulation efficiency of approximately 3- and 10-fold, respectively. Another challenge restricting the practical application of Fn nanoparticles in drug delivery systems is protein aggregation, which results in low production efficiency during the drug encapsulation process. Based on a recent study, the use of high hydrostatic pressure (HHP) as a novel approach enhanced the loading of the anticancer drug, DOX, into Fn nanoparticles [46]. At pH 5.5 and a pressure of 500 MPa, the drug was efficiently loaded into the nanocarrier. Furthermore, the addition of 20 mM arginine completely inhibited DOX/Fn aggregation, resulting in monodisperse particles with a protein recovery rate of 100%.

Many researchers have attempted to provide Fn systems with site-specific delivery and enhanced stability [47,48]. Several chemical or genetic engineering approaches have been applied to construct new Fn fusions with unique properties. For instance, Shan et al. developed a Fn nanoparticle vector for the targeted delivery of CpG oligodeoxynucleotides (CpG ODNs) to M2-type Tumor-associated macrophages (TAMs). CpG ODNs have been encapsulated inside Fn nanocage surfaces genetically linked with a murine M2 macrophage-targeting peptide (M2pep) [23]. Upon intravenous injection of M2pep-Fn-CpG nanoparticles, M2 TAMs were repolarized to the M1 type, and tumor growth was inhibited. According to another study, Luc-linker@Fn was identified as an optimal preclinical bioluminescent probe for the study of nanodrug targeting of tumor cells via chemical conjugation of luciferin with human Fn [24]. Luc-linker@Fn has been demonstrated to cyclize once internalized into luciferase^+^ 4T1 cells due to the reductive environment caused by glutathione. Falvo et al. also constructed an antibody-Fn-loaded drug conjugate to target melanoma cells [25]. Briefly, Fn nanoparticles loaded with 50 molecules of cisplatin were conjugated with a monoclonal antibody (mAb), Ep1. This mAb-derivatized Fn–Pt–Ep1 nanoparticle performed 25 times better in melanoma cells than the HFn–Pt–Ep1 nanoparticle alone, which inhibited thymidine incorporation more efficiently.

**Figure 1:**
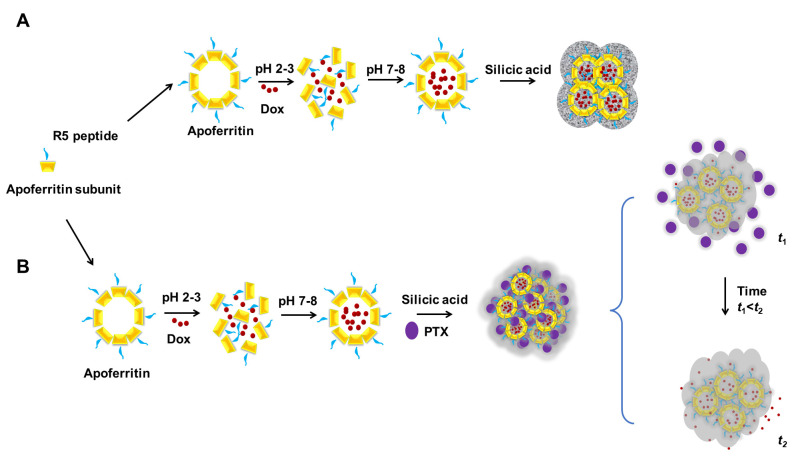
Schematic for the preparation of the engineered ferritin nanoparticles and development of dual drug delivery systems. The pH-triggered dissociation and association mechanisms of the engineered Fn molecules were used for drug loading. The silica-forming peptide (silaffin R5) tagged Fn fusion protein was self-entrapped in biosilica, acting as a nanocarrier for (**A**) one-drug or (**B**) two-drug delivery. (**A**): Reproduced with permission from [26], Copyright 2018 Elsevier Ltd. (**B**): Reproduced with permission from [27], Copyright 2019 The Korean Society of Industrial and Engineering Chemistry (Elsevier).

Interestingly, the Pack group established an innovative system for drug delivery by integrating two biocompatible approaches, Fn nanocages and bioinspired silicification (Figure 1) [26]. As a result, the surface of Fn nanoparticles was genetically modified by presenting a short silica-forming peptide (SFP) on its surface, which could direct the deposition of biosilica around chimeric Fn fusion proteins [49,50]. The fabrication process was further controlled to produce sub-50 nm bioinspired silica/Fn nanoparticles [51]. Subsequently, the constructed SiO_2_/Fn-SFPs nanoparticles showed high loading of DOX with pH-responsive drug release patterns. This biocompatible system was further optimized to develop a dual-drug delivery system by loading two molecules separately into the core (protein cage) and outer shell (silica matrix) (Figure 1) [27]. In this process, the first anticancer drug, DOX, was encapsulated inside the constructed Fn nanocage. The second drug, PTX, was loaded during the biomineralization process, resulting in the production of SiO_2_(PTX)/Fn(DOX) [27]. Interestingly, PTX loaded in the silica shell was released short-term, whereas DOX loaded in the Fn core was released in long-term. These findings indicate that the stabilization of Fn nanocages with a biosilica coat could be an efficient approach for enhancing the loading and controlling the delivery of a single or combination of drugs.

### 2.2. Application of Engineered Vault-Based Nanoparticles in Therapeutics Delivery

**Figure 2:**
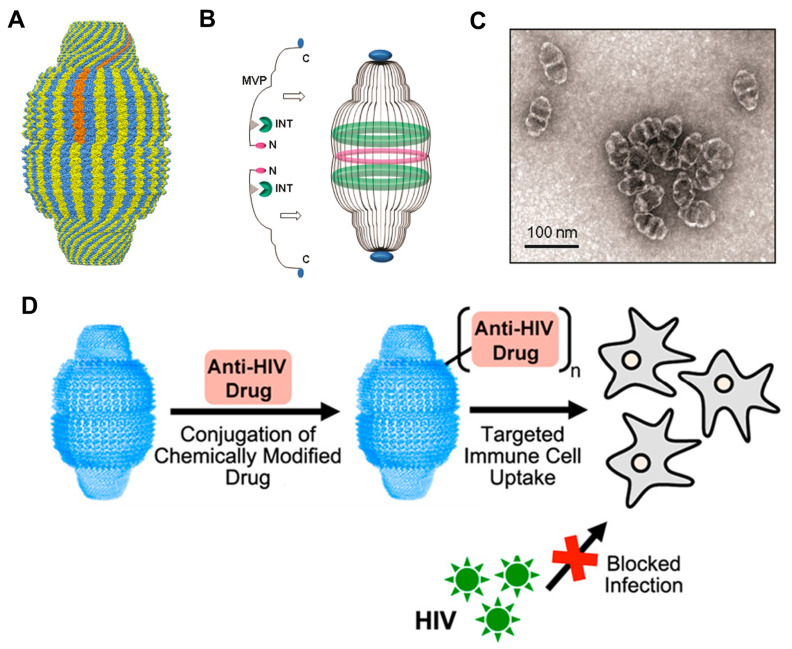
Structure of vault nanoparticles. (**A**) Side view showing the arrangement of major vault protein (MVP) chains that form the vault surface. (**B**) Strategy for fusion protein packaging into multifunctional vaults using the MVP interaction (INT) domain attachment. (**C**) Transmission electron microscope (TEM) image of recombinant pVI-MVP vault particles. (**D**) Schematic illustration of the conjugation of the vault nanoparticles with antiretroviral drugs and its inhibition of HIV-1 infection in a human cell line. (**A**): Reproduced with permission from [53], Copyright 2013 American Chemical Society. (**B**,**C**): Reproduced with permission from [28], Copyright 2011 American Chemical Society. (**D**): Reproduced with permission from [30], Copyright 2019 American Chemical Society.

Vault nanoparticles are among the largest known sub-100 nm ribonucleoprotein complexes (Figure 2A) [52,53]. Vaults are involved in various cellular processes, including innate immunity, nuclear-cytoplasmic transport, drug resistance, nuclear pore assembly, and cell signaling, with intriguing applications as nanodevices for the delivery of multiple cargos [54]. Vault proteins, which have a mass of approximately 13 MDa, form three-dimensional nanoparticles (67 nm × 40 nm × 40 nm in size), and are abundant and conserved in most eukaryotes [55]. In native vaults, three proteins are present in multiple copies: vault poly(ADP-ribose) polymerase (193 kDa, VPARP), vault telomerase-associated protein (290 kDa, TEP1), and major vault protein (100 kDa, MVP) [56]. The most recent approaches for recombinant vault production only exploit genetically expressed MVP because it accounts for 75% of the natural vault protein mass and is adequate to create vault nanoparticles [57]. Vault particles are promising therapeutic nanocarriers for bioactive drug encapsulation owing to their unique properties, such as self-assembled proteinaceous nanostructures with a large internal volume (5 × 10^4^ nm^3^) [29]. In addition, the ability of these proteins to dissociate into half in a low-pH environment could enhance their usefulness for drug delivery [58].

Vault proteins are biomedically useful because of their non-immunogenicity and structural stability [57]. Accordingly, these proteins are used as precise nanocarriers for the delivery of several immunogenic proteins, drugs, and other biomolecules (Table 1) [59]. Han et al. demonstrated the possibility of producing functional vault nanodisks via the genetic fusion of the MVP domain with a membrane lytic peptide (pVI) derived from adenovirus protein VI to examine their escape from the endosomal compartment [28]. Efficient delivery of vaults to the cytoplasm was achieved via the direct targeting of pVI-vault fusion to particular cell surface epidermal growth factor receptors. This strategy can be extended to present multifunctional exogenous proteins on vault nanoparticles via fusion with the MVP interaction domain (INT) (Figure 2B,C). Additionally, genetically engineered vault nanoparticles loaded with CCL21, a lymphoid chemokine, elicit antitumor activity by inhibiting lung cancer growth during targeted delivery [29]. Interestingly, CCL21/vault complex administration increased leukocyte infiltration recruitment and slowed tumor development in mice with lung cancer [29].

Recently, Fulcher et al. found that the cells critical to the pathogenesis of human immunodeficiency virus type 1 (HIV-1) infection and transmission are natural targets of the uptake of vault proteins (Figure 2D) [30]. These researchers employed a novel approach with recombinant human vault nanoparticles for drug delivery by conjugating antiretroviral drugs to the recombinant vault nanoparticles. As a result, a targeted drug delivery system was developed to prevent the infection of key cell types involved in HIV-1 transmission. Recombinant vault nanoparticles and nanodisks have been applied for targeted delivery in several therapeutics, such as mCherry and epidermal growth factor [31], bryostatin 1 and amphotericin B [55], and enhanced green fluorescent protein (EGFP) cDNA and saporin [60]. Notably, genetically modified vaults have been demonstrated to be non-toxic, efficient, and effective nanoparticles for the targeted delivery of biomaterials.

As vaults have gained more attention for targeted therapeutic delivery, several scientists are interested in further developing this delivery system by enhancing their production and purification systems and improving their stability. Galbiati et al. explored the possibility of developing efficient production and purification methods for vault fusion. Previously, a baculovirus expression vector system was employed for this development [61]. However, vault purification requires a two-step process: dialysis followed by size-exclusion chromatography. Martin et al. explored the possibility of developing faster and more efficient strategies for vault production and purification [32]. Instead of the current tedious and time-consuming protocol using insect cells [62], the researchers employed the expression of His-tagged MVP (MVP-H6) in a human cell line, in which the expressed protein could be purified using one-step immobilized metal affinity chromatography (IMAC). The co-expression of MVP-H6 and INT-tagged cargo proteins allows the spontaneous bioengineering of self-assembled vault nanoparticles and their cargo loading within cell factory production. This approach could render a straightforward procedure for the easy and fast biofabrication of recombinant vaults that are ready-to-use in drug delivery systems.

Recently, Wang et al. introduced new functionalities on the surfaces of vault nanoparticles by employing a bioinspired silicification approach to produce a more stable structure [63]. The engineered recombinant vault nanoparticles loaded with bioactive proteins enabled the deposition of a protective biosilica layer around them, which could expand the application of vault technology in biosensors and drug delivery systems.

### 2.3. Application of the Nano-Compartments of the Engineered Encapsulin in Therapeutic Delivery

Encapsulins are engineered and robust proteins that form hollow nanocapsids, making them desirable nanocarriers for bioactive therapeutic delivery. Encapsulins are found in many archaea and bacteria [64]. Encapsulin from the thermophilic bacterium, *Thermotoga maritima*, is a well-studied model consisting of 60 copies of identical 31 kDa monomers with interior and exterior diameters of 20 and 24 nm, respectively [65].

Genetically engineered encapsulin nanocages have been employed for anticancer therapeutic delivery (Table 1) [33]. In fact, the human hepatocellular carcinoma (HCC)-cell binding domain (SP94, SFSIIHTPILPL) was genetically displayed on the surface of the encapsulated nanocage, effectively targeting HepG2 cells. The anticancer prodrug, Aldoxorubicin (AlDOX), was loaded onto the SP94/Encapsulin complex through a chemical modification approach. The killing effectiveness of the engineered encapsulin-loaded AlDOX complex was comparable to that of free AlDOX but did not exhibit any intrinsic cytotoxicity. In another study, a genetically functionalized encapsulin protein nanocage was employed as a squamous cell carcinoma (SCC-7) targeting optical nanoprobe [34]. The encapsulin protein was fused with an Fc-binding peptide (FcBP sequence with linker GGGGGGDCAWHLGELVWCTGGGGG). Fluorescence imaging demonstrated that the labelled FcBP-encapsulin was specifically attached to the SCC-7 cell line, but not to MDA-MB-231, HepG2, and HeLa cells. As engineered encapsulin allows the simultaneous incorporation of therapeutic reagents targeting ligands and diagnostic probes, there are prospects to construct multifunctional nanoplatforms for drug administration or theranostic systems.

The Designed Ankyrin repeat protein (DARPin9.29), an antibody-mimicking protein, was displayed on the encapsulin for targeted drug delivery [35]. Using the bacterial expression system, an engineered flavin-binding protein mini-singlet oxygen generator (MiniSOG) and the encapsulin-fusion protein, DARPin9.29 fusion protein, were coproduced and purified from *E. coli* cells. The encapsulin-DARPin_miniSOG nanocompartment effectively binds to HER2-positive breast cancer cells and causes apoptosis, indicating the specificity and functionality of the system.

Cornelissen et al. recently explored the genetic surface modifications of encapsulin from *Brevibacterium linens* (Bl) and *T. maritima* (Tm) [66]. A His-tag loop, a well-known purification tag, was inserted at two positions on the Tm encapsulin surfaces and the C-terminal of the Bl encapsulin. Interestingly, the multi-modified Tm encapsulin could achieve 240 functionalities at the surface of the cage, which resulted from four possible modifications per subunit of the protein. Despite maintaining the integrity of the cage, this study revealed that surface modifications affected the cage structure and caused long-term stability differences. In addition to offering improved short-term stability of encapsulins, the researchers demonstrated sporadic long-term stability in some samples.

**Figure 3:**
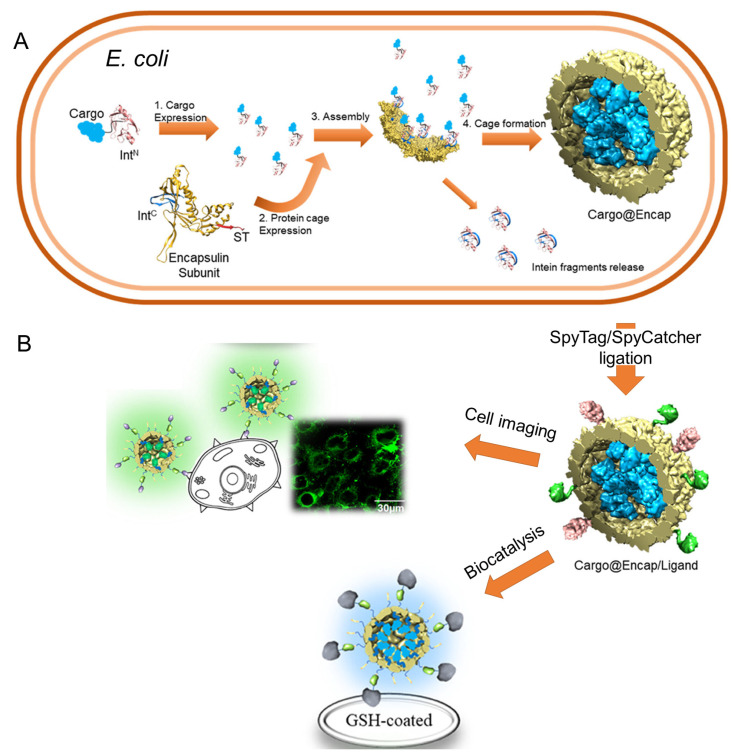
Schematic illustration of the engineered encapsulin self-assembled nanostructures as integrated nanoplatforms with selective functionalization of their interior and exterior surfaces. (**A**) Intein-mediated ligation during the expression of the fusion proteins results in self-encapsulation of the cargo molecules into the interior space of Encap. (**B**) Use of the SpyTag/SpyCatcher ligation system to functionally display the ligands on the Encaps. The bioengineered nanostructures showed potential applications in targeted cell imaging, multilayered nanostructure formation, and biocatalysts. (**A**,**B**): Reproduced with permission from [36]. Copyright 2021 American Chemical Society.

In a recent study, both termini of *T. maritima* encapsulin were genetically modified by introducing a SpyTag and split-C-intein (IntC) fragment into the exterior and interior surfaces, respectively (Figure 3) [36]. Using the SpyTag/SpyCatcher ligation system, a variety of therapeutic proteins were displayed on encapsulin (Encaps) surfaces via fusion with SpyCatcher protein, such as an epidermal growth factor receptor (EGFR)-overexpressing cancer cells-targeting affibody (SC-EGFRAfb), dimerizing ligands (FRB-SC and FKBP12-SC), and a glutathione binder (GST-SC). Additionally, split intein-mediated protein ligation led to the successful encapsulation of various protein cargoes, including NanoLuc luciferase (Nluc), Nluc-miniSOG, and EGFP, via individual fusion with split-N-intein (IntN) into Encaps to form Cargo@Encap. The applications of the constructed protein nanostructures in the biomedical field have been investigated. The (EGFP)@Encap/EGFRAfb complex served as a target-specific fluorescent imaging probe to selectively visualize specific cancer cells. Furthermore, immobilization of Nluc@Encap/GST nanoparticles on a glutathione-coated support enhanced the long-term stability of the encapsulated enzyme. These results demonstrate that targeted functionalization of the encapsulin’s exterior surface and interior cavity could enable the generation of novel protein-based nano-biomedical tools.

Encapsulin-based nanoparticles have been successfully used in vaccine applications. Lagoutte et al. produced encapsulin nanocompartments in *E. coli,* which were employed for rational vaccine design [67]. Although the green fluorescent protein (GFP) was packaged in the internal space of *T. maritima* encapsulated in nanoparticles, surface display of the matrix protein 2 ectodomain (M2e) of influenza A virus was achieved. Engineering the encapsulating surface with M2e had a positive effect on heterologous protein-loading capacity in the interior space, indicating that the stiffness might be increased by a crucial elastic deformation induced by the surface-displayed protein. Furthermore, mouse immunogenicity studies revealed antibody responses against loaded cargo proteins and surface epitopes. According to these findings, rational vaccine formulations can be prepared using encapsulin-modified nanoparticles that package and display antigens simultaneously.

### 2.4. Application of Small Heat Shock Proteins (sHSPs) in Therapeutic Delivery

Despite being known as protein chaperones for many years [68], small heat shock proteins (sHSPs) have attracted considerable attention in the drug delivery field [69]. sHSPs are ubiquitous molecular chaperones that prevent the accumulation of proteins during heat shock [70]. sHSPs contribute to cell survival and death by preventing protein aggregation. The application of an sHSP nanocage from *Methanococcus jannaschii* (Mj-sHSP) as a targeted drug vehicle was investigated (Table 1). Douglas et al. recently explored the application of sHSP for the delivery of the anticancer drug, DOX. The inner surface of Mj-sHSP was genetically modified to include cysteine residues, and pH-sensitive linkers were used to covalently connect DOX molecules to the cysteine residues. In acidic environments, the hydrolysis of hydrazone linkages allows the selective release of anticancer drugs [37]. Additionally, RGD-4C (CDCRGDCFC), a tumor vasculature-targeting peptide, was incorporated into the external surface of the Mj-sHSP cage using a genetic approach [38], which produced modified Mj-sHSP cages with cell-specific targeting capabilities. Similarly, the SP94 peptide was functionally displayed on the surface of small heat shock protein (HspG41) nanoparticles using a genetic engineering approach to selectively target HCC cells [39]. Interestingly, the ability of the DOX-loaded HspG41C-SP94 nanocarrier to selectively target HCC cells significantly decreased its cytotoxicity toward normal hepatocytes, while maintaining its cytotoxic effects against Huh-7 HCC cells.

**Figure 4:**
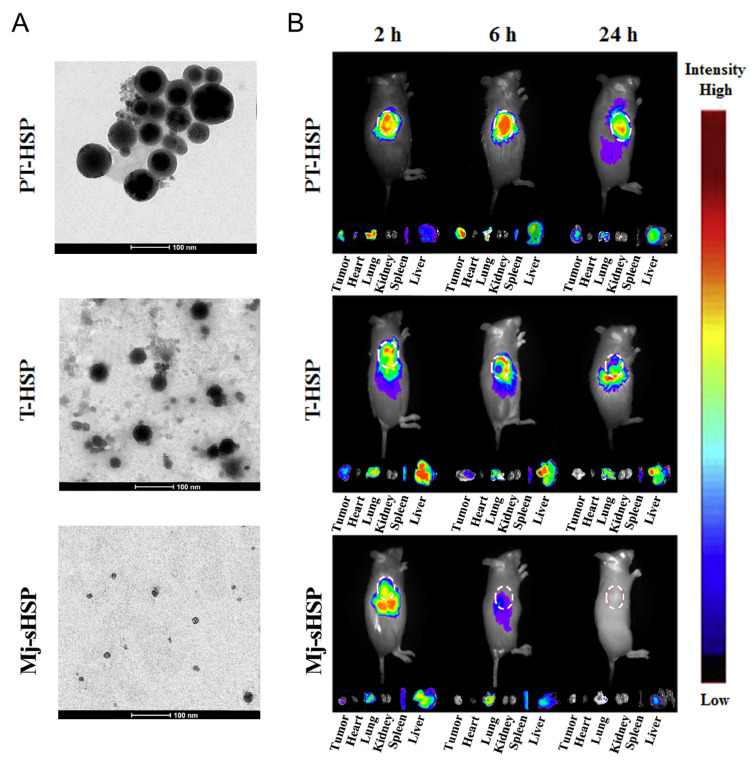
(**A**) TEM images of the fusion proteins, Mj-sHSP, T-HSP, and PT-HSP nanoparticles. (**B**). In Vivo imaging study of Cy5.5-labelled protein nanoparticles injected intravenously into H22 tumor-bearing mice. (**A**,**B**): Reproduced with permission from [40]. Copyright 2020 Acta Materialia Inc. Published by Elsevier Ltd.

A conjugation of Mj-sHSPs with a cell-penetrating peptide (transactivator of transcription, TAT) self-assembled into T-HSP nanoparticles was used to target the tumor microenvironment (TME) (Figure 4) [40]. The self-assembled nanoparticles were further coated with PEG/N-(2-aminoethyl)piperidine-hyaluronic acid (PAHA) as a pH-sensitive molecule. Interestingly, the loaded PTX in the (PTX) PT-HSP complex was released in a pH-controlled manner, with negligible release under physiological pH conditions. However, under TME mimicking conditions (overexpressed hyaluronidase (HAase) at acidic pH), the PAHA coat was degraded, which triggered drug release. Additionally, the ability of PT-HSP nanoparticles to internalize the microenvironment of normal cells was shielded and recovered in cancerous cells. Compared with the uncoated control, in vivo imaging revealed that the PT-HSP nanoparticles displayed outstanding tumor-targeting capabilities.

### 2.5. Application of Engineered Elastin-like Polypeptide Nanoparticles in Therapeutics Delivery

Elastin-like polypeptides (ELPs) are biomaterials composed of hydrophobic repeats of tropoelastin [71]. ELPs consist of the short repeating peptide motifs, VPG*X*G, where *X* is a guest residue that is any amino acid except proline [72,73]. ELPs can impart and precisely tune many interesting properties, such as reversible phase separation in aqueous solutions, by changing the identity of X [74,75,76]. Additionally, ELP block copolymers are made by genetically linking hydrophilic blocks to hydrophobic blocks, such as [(VPGSG)_48_(VPGIG)_48_] [77] or (VPGVG)n-(VPGKG)m [78] which are intriguing applications for the production of ELP polymers with distinct characteristics based on the modifications of the guest residue. These block copolymers have been demonstrated to form stable nanoparticles with diameters as small as 100 nm, depending on the differences in transition properties between hydrophilic and hydrophobic blocks [79,80]. Interestingly, thermoresponsive self-assembled coacervates are formed via phase separation of the ELP biopolymers at a lower critical solution temperature (LCST) [81,82]. A reversible LCST transition occurs when the solution temperature is lowered below the transition temperature (T_t_), allowing the ELP coacervate to resolubilize. Therefore, the phase transition of ELP could be triggered by various other stimuli, including pH, salts, or the number of repeats in the guest residue at position X, in addition to the temperature [83,84]. Consequently, genetically engineered ELP nanoparticles have become popular in drug delivery approaches (Table 1) [85].

Han et al. explored the capability of a biomineralization approach to enhance the delivery properties of genetically engineered ELP micelles loaded with drugs [86]. In this study, the ELP sequence ([VPGAG]_40_) was genetically fused with the silica-forming peptide silaffin R5 at the N-terminus to target silica deposition and a cysteine-containing (CGG)_8_ sequence at the C-terminus for drug conjugation. By interacting with drug molecules (DOX), the thiol group of cysteine provided sufficient amphiphilicity to induce ELP-DOX conjugate self-assembly into spherical micelles. In addition, the silaffin R5 peptide displayed on the surface of the assembled ELP/DOX complex accelerated the deposition of silica around the protein-drug micelles, resulting in a uniform hybrid biomaterial composed of silica/ELP-DOX. Interestingly, DOX was released from the biomineralized protein micelles in a pH-dependent manner.

For the specific delivery of ELP nanoparticles to cancer cells, the targeting and internalization of molecules on ELP particle surfaces can be achieved via a genetic engineering approach. Kobatake et al. constructed an ELP fused with a poly aspartic acid tail (ELP-D) and internalizing RGD (iRGD) for use in targeted drug delivery [42]. The engineered protein fusion produced stable nanoparticles owing to the coacervation of the charged poly aspartic acid chain with ELP at high temperatures. The iRGD-displayed engineered fusion nanoparticles were applied for the delivery of PTX to targeted cancer cells via integrin binding, cell internalization via the NRP-1 pathway, and induction of cell death. Additionally, Lui et al. developed a robust hybrid nanosystem combining ELPs with mCherry to precisely deliver an anticancer peptide (PCK3145) to colon adenocarcinoma [41]. ELP nanoparticles coated with the GE11 peptide (YHWYGYTPQNVI) can bind to colon tumors expressing EGFR in vitro and in vivo. Compared with untargeted nanoparticles, treatment with GE11 and PCK-ELPs-mCherry resulted in a 2-fold increase in apoptotic cell death. These versatile nanoparticles effectively inhibited tumor growth in mice harboring colon cancer xenografts.

**Figure 5:**
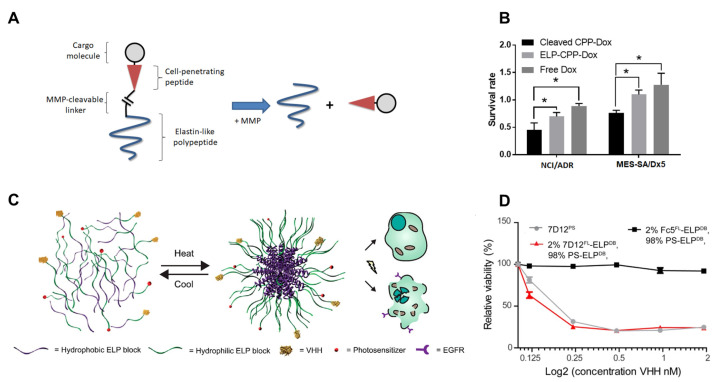
Elastin-like peptide (ELP)-based drug delivery system using (**A**) the fusion of ELP with (MMP) cleavable linker and cell-penetrating peptide (CPP), and drug molecules. (**B**) Cytotoxicity of the cleaved CPP-DOX compared with the ELP-CPP-DOX (* *p* < 0.05). (**C**) Schematic of photodynamic therapy based on the fusion of ELP with VHHs. The self-assembled nanostructure was produced by mixing amphiphilic diblock ELP with functionalized ELP. (**D**) Relative cell viability after incubation and illumination with two distinct VHH-ELP fusion proteins, 7D12^FL^-PS-ELP^DB^ micelles (7D12 used as the VHH against EGFR) and Fc5^FL^-PS-ELP^DB^ micelles (Fc5 used as an unrelated VHH control), or free 7D12^PS^ (control). (**A**,**B**): Reproduced with permission from [88], Copyright 2021 MDPI. (**C**,**D**): Reproduced with permission from [89], Copyright 2017 American Chemical Society.

Multidrug resistance (MDR) is one of the most influential factors contributing to chemotherapy failure [87]. The efficacy of anticancer drugs decreases over time as cancer cells become drug-resistant. Therefore, new strategies for overcoming MDR are urgently required. Dragojevic et al. established a macromolecular drug delivery system based on ELP to precisely deliver DOX to targeted cells by inhibiting drug resistance [43]. This system consists of a multi-biomacromolecule, cell-penetrating peptide (CPP), ELP, and pH-sensitive cleavable linker to release the conjugated DOX. A cysteine residue was included in the ELP fusion, which was then coupled to DOX derivatives with thiol-maleimides. Compared with unconjugated DOX, DOX delivered by ELP fusion has comparable toxicity to sensitive and drug-resistant cell lines. Similarly, conjugation of CPP and ELP with a matrix metalloproteinase (MMP) substrate linker for DOX cleavage increased cell penetration by 4-fold and doubled the killing of resistant cells compared to free DOX and ELP-DOX (Figure 5A) [88]. Owing to the pharmacokinetics and targeting benefits conferred by conjugation to ELP, these biopolymers can overcome DOX resistance in vivo.

ELP has been demonstrated to significantly affect the short blood circulation time of peptide drugs. In fact, the fusion of ELP with a recombinant llama heavy-chain antibody fragment (VHH) against EGFR resulted in multivalent particles with a hydrodynamic radius of 24.3 nm and retention of the total EGFR-binding capacity (Figure 5B) [89]. Additional light-induced cell death was triggered by the incorporation of a photosensitizer into the self-assembled VHH-ELP. Interestingly, this process could improve therapy and diagnostics using targeted nanomedicine by controlling the incorporation of different functional modules.

Intriguingly, several antibody-ELP conjugates have been used to construct multivalent nanoparticles. For instance, the fusion of amphiphilic immune tolerant ELP with the single-chain variable fragment (scFv) of αPD-1 checkpoint inhibitor, which self-assembled into nanoparticles, had a greater affinity for binding to αPD-1 than the intact αPD-1 antibody or the monomeric scFv [44]. Interestingly, a novel worm-like structure was fabricated via the genetic fusion of ELP with an anti-CD99 scFv [45]. When tested for its ability to treat leukemia in mice, the engineered nanoform of the ELP-fused CD99 nanoparticles was circulated for 16 h and was retained for 21.3 h. Furthermore, the leukemia burden in the treated group was significantly reduced, and survival was prolonged. Conrad et al. used a plant expression system [90] to produce antibody-ELP fusion proteins [91]. Fusion of ELP with an anti-human tumor necrosis factor (TNF) nanobody extended the systemic circulation half-life, and induced a 24-fold increase in the serum half-life of the anti-TNF-ELP fusion.

## 3. De Novo Design of Protein-Based Nanoparticles for Efficient Drug Delivery

The current tailored engineering tools and processing capabilities involving directed evolution and genome editing could increase the number of protein nanocarriers available to reach the market [92,93]. Interestingly, custom proteins with distinct shapes that may not be possible in nature can be selected via directed evolution with de novo protein design [94]. Modular building concepts can be used to fold proteins correctly in programmable ways and enable their assembly into complex architectures [95].

Computationally designed approaches have been developed to produce precise protein assemblies [96]. The capability of computational techniques to design highly homogenous self-assembled nanoparticles could open up new routes for vaccine design and drug delivery [97]. For instance, Baker and colleagues developed an algorithm to arrange protein building blocks symmetrically and determine the lowest-energy protein-protein interfaces for self-assembling protein architectures [98,99]. Using Rosetta Design, possible interfaces that resemble natural nanostructures could be generated in a high-throughput screening manner. A recent program developed by Baker simulated the co-assembly of multiple copies of different subunits into specific nanostructures [100]. This team created five nanocage structures with 24 subunits each, proving their theoretical work through experimentation. Interestingly, these structures have an energy-compliant structure owing to the symmetry and multiple symmetry axes aligning dimeric and trimeric subunits. This study demonstrated the practicality of designing de novo nanomaterials using a two-compartment model that precisely aligns with the computed models. 

Similarly, a unique self-assembled polypeptide nanocage, known as self-assembled peptide caGEs (SAGE), was de novo designed by Woolfson and colleagues via merging of heterodimeric and homotrimeric coiled coils in a non-linear configuration [101]. The SAGE nanostructure is a hexagonal lattice of two complementary 3-fold symmetric peptide hubs (peptide building blocks, hubs A and B), resulting in the self-assembly of hollow spherical particles with a diameter of approximately 100 nm; these particles are suitable for drug encapsulation and controlled release.

**Figure 6:**
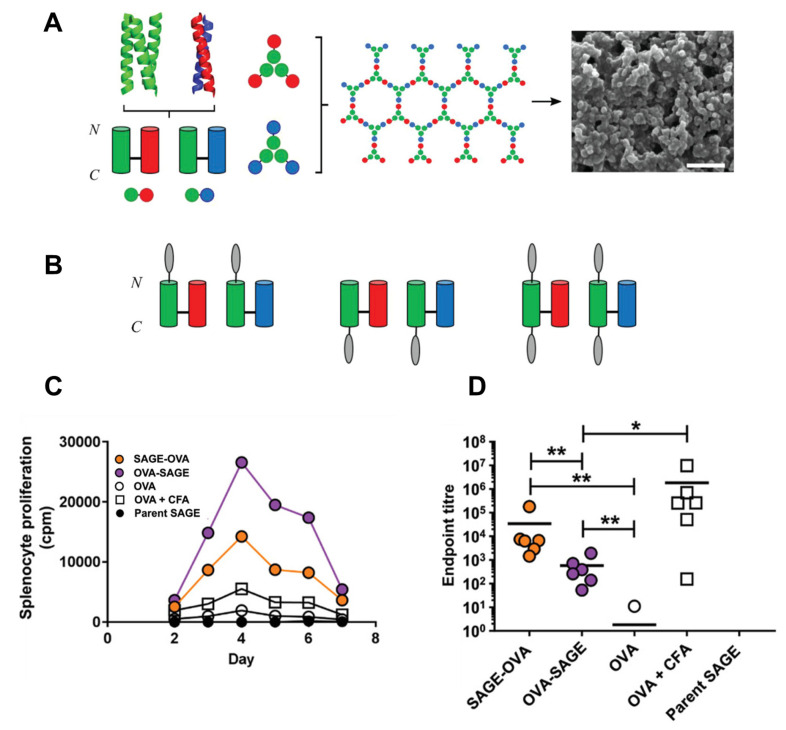
(**A**) Schematic of the self-assembly of SAGE nanostructures. Complementary hubs are formed by linking the heterodimer to the homotrimer. The hubs are self-assembled into a lattice with a particle size of ~100 nm, as indicated by scanning electron microscope (SEM). The scale bar of the SEM image is 1 µm. (**B**) Incorporating distinct model antigenic peptides (gray ovals) into hubs as single or double fusions. (**C**) Proliferation of murine splenocytes stimulated with OVA peptide after booster immunization. (**D**) Anti-OVA antibody production was monitored by EPT ELISA using antigenic OVA peptide. * *p* < 0.05 and ** *p* < 0.01. (**A**–**D**): Reproduced with permission from [102]. Copyright 2019 WILEY-VCH Verlag GmbH & Co. KGaA, Weinheim.

SAGE nanostructures can be further functionalized via chemical or genetic approaches with other proteins or peptides to promote cell-specific uptake. For instance, genetic fusion of the fluorescent protein GFP to the N- or C-terminus SAGE peptide hubs enables self-assembly into protein-SAGE nanospheres (named pSAGE) [103]. Furthermore, multiple different proteins fused with the SAGE hub domain were assembled and simultaneously integrated into the same particle, creating new routes for the future use of these biomaterials as drug delivery and vaccination platforms. In another study, decoration of the SAGE nanostructure with small, charged peptides (tetra-lysine or tetra-glutamic acid tags) was carried out to modulate the endocytic uptake of the particles by HeLa cells. Interestingly, the peptide charge had an impact on the uptake efficiency of the particles in mammalian cells. Surface decoration with a positively charged peptide (tetra-lysine) increased endocytic uptake, whereas decoration with a negatively charged peptide (tetra-glutamic acid) hindered uptake. Further control over the cellular uptake of SAGE nanoparticles can be achieved by adjusting the stoichiometry of the tetrapeptide extensions.

The potential application of engineered SAGE nanoparticles as modular scaffolds for antigen delivery has been investigated (Figure 6) [102]. Based on in vivo mouse assays, the prepared nanoparticles, through the fusion of the antigens to either the N- or C-terminus of SAGE nanostructures, were found to be non-toxic. Moreover, cellular uptake of C-terminal-functionalized SAGE nanoparticles with the model tetanus toxoid antigen (TT) was enhanced. The response to the TT-functionalized SAGEs was also found to be more potent than that of the free TT peptide. However, the fusion of TT at the N-terminus of the SAGE nanostructure reduced the uptake of SAGE by cells, impairing the proliferation of CD4^+^ T cells. The comparison between SAGE-functionalized C-terminally with ovalbumin antigen (OVA) and those functionalized N-terminally with OVA indicated a higher CD4^+^ T cell proliferation response in contrast to a lower antibody response in SAGE-functionalized at the C-terminal with OVA (Figure 6B–D). Different antigens can be displayed in the same self-assembled SAGE nanoparticles by varying the mixing ratios and types of antigen-fused peptides to boost immunogenicity. Although these results are encouraging, more studies are necessary before SAGE vaccine candidates can be used in the clinic.

**Figure 7:**
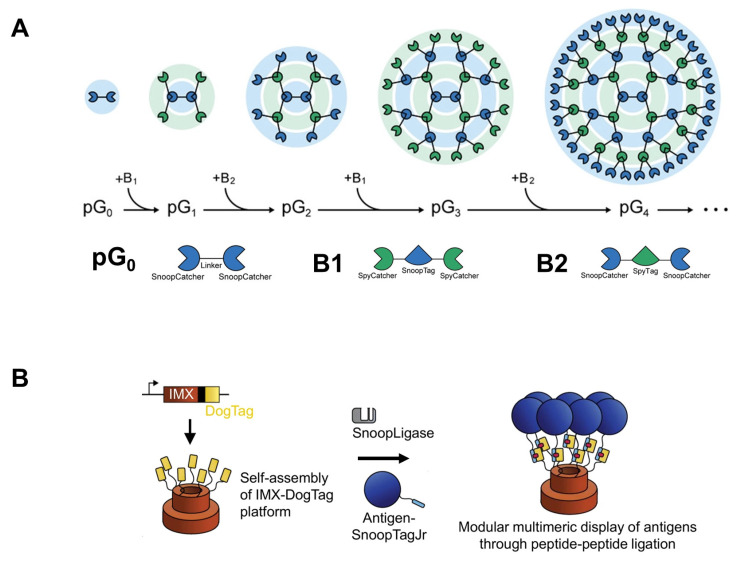
(**A**) Graphical representation of the stepwise growth of protein dendrimer nanostructures through the addition of the building fusion polypeptides, B1 and B2, to the core protein (pG0). (**B**) An overview of vaccine assembly mediated by SnoopLigase. IMX-DogTag nanoparticles are produced via spontaneous oligomerization. SnoopLigase stimulates the formation of isopeptide bonds between antigen-SnoopTagJr and IMX-DogTag. (**A**): Reproduced with permission from [104], Copyright 2021 Advanced Science published by Wiley-VCH GmbH. (**B**): Reproduced with permission from [108], Copyright 2019 Springer Nature.

The programmable assembly of protein nanoparticles could provide a route for the construction of innovative functional nanomaterials for potential applications in nanomedicine. Bae et al. applied a bottom-up method to construct supramolecular assemblies of protein nanoparticles with programmable size and valency [104]. The protein was assembled into dendrimer-like nanostructures, called “protein dendrimers”, using three genetic fusion proteins (one core and two building-block proteins) (Figure 7A). The core protein is a genetically fused dual-tandem SnoopCatcher dendrimer that represents the zeroth generation (pG0). The two building blocks (B1 and B2) are repeats of SpyCatcher or SnoopCatcher connected with SnoopTag or SpyTag, respectively. Covalent isopeptide bonds are spontaneously established between the catcher and tag, which shares orthogonal pairs of these fusion proteins [105,106]. The protein dendrimer was grown successively from pG0 (27 kDa) to pG1, pG2, pG3, and pG4 (94, 216, 483, and 959 kDa, respectively). Compared with a monomeric design, multivalent protein dendrimers considerably enhance their binding affinity for a target. Accordingly, the functionalization of recombinant protein dendrimers with gelonin, a plant-derived N-glycosidase that acts as a cytotoxic protein cargo, induced a higher cytotoxic effect on cell viability. Owing to the biodegradability and biocompatibility of protein dendrimers compared with metal oxide nanoparticles, this approach can be effectively employed in the construction of advanced protein assemblies for biotechnology and nanomedicine.

Similarly, multimerizing coiled-coil IMX313 (a hybrid version of the chicken complement inhibitor C4b-binding protein) [107] was genetically fused with SpyCatcher and SnoopCatcher to produce SpyCatcher-IMX-SnoopCatcher [105]. The engineered fusion proteins served as a modular platform in which antigen fusions with either SpyTag or SnoopTag could be simply multivalent and displayed on the opposite sites of self-assembled protein nanoparticles. The multivalent surface decoration of SpyCatcher–IMX–SnoopCatcher protein nanoparticles with malarial Pfs25 and Pfs28 markedly enhanced the antibody response to both antigens by approximately 100-fold. In fact, these nanoparticles enable a robust antibody response after only a single immunization, indicating the potential application of these biomaterials in the biomedical field.

A recent study revealed the application of SnoopLigase, an engineered protein derived from *Streptococcus pneumoniae* adhesin, to enable isopeptide bond formation between DogTag and SnoopTagJr (Figure 7B) [108]. Owing to SnoopLigase, SnoopTagJr-tagged antigen could be conjugated to the IMX-DogTag complex, resulting in multivalent antigen presentation. Interestingly, noopligase-mediated self-assembly of IMX fusions enhanced the antibody response to two blood-stage malarial proteins (PfEMP1 and CyRPA) in a prime-boost model.

Biomimetics of DNA origami nanostructures [109], modular de novo design of self-assembled polyhedral protein cages, triangular prisms, four-sided pyramids, and tetrahedrons have been efficiently constructed in vitro and in vivo using protein coiled-coil (CC) dimers as building blocks [110]. The self-assembly of tetrahedral CC protein-origami (CCPO) structures displayed properties similar to those of natural proteins, such as high stability and folding kinetics. The potential mechanism for producing CCPO nanocages in vivo in bacteria, mammalian cells, and mice without causing inflammation suggests that CCPO nanocages can be used to deliver drugs, vaccines, and other medical applications.

**Figure 8:**
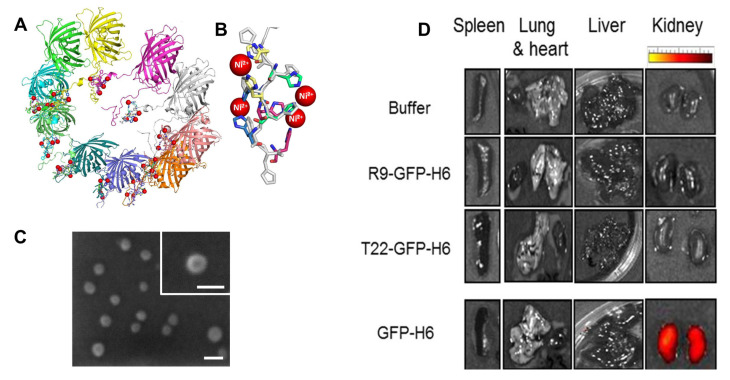
(**A**) Self-assembly of the T22-GFP-H6 fusion protein through the His-Ni^2+^ interaction model. (**B**) Details of the His residues superimposed on the N-terminus of T22-GFP-H6 monomers. (**C**) SEM images of GFP fusion nanoparticles. Scale bar indicates 20 nm. (**D**) Stability and biodistribution of the GFP fusion proteins. GFP fluorescence signals were observed in mouse organ sections and kidneys two hours after iv administration of GFP fusion proteins. (**A**,**B**): Reproduced with permission from [114], Copyright 2018 Acta Materialia Inc. Published by Elsevier Ltd. (**D**): Reproduced with permission from [113], Copyright 2014 American Chemical Society.

A de novo oligomerization approach was used to produce self-assembled recombinant protein nanoparticles based on the fusion of target therapeutic proteins with dual peptide tags, a cationic peptide tag, and a polyhistidine peptide tag [111]. Both tags promote the assembly of fusion proteins into nanoparticles (10–80 nm), which are stabilized by non-covalent interactions and the coordination of divalent cations [112]. Based on this strategy, several protein-based nanoparticles have been developed and applied for therapeutic delivery. N-terminal cationic peptide fusions, T22, and arginine-rich R9 (used in nanomedicine for C-X-C chemokine receptor type 4 (CXCR4) cell targeting and brain targeting, respectively) fused to 6xHis-tagged GFP (GFP-H6) resulted in the production of fluorescent protein nanoparticles with sizes of 13 nm and 20 nm, respectively (Figure 8) [113]. Based on renal clearance and biodistribution analyses of GFP nanoparticles, the nanoparticles maintained their architectural stability over time, enabling systemic circulation and tissue targeting.

The potential applications of GFP fusion protein nanoparticles in therapeutic delivery were further extended by terminal fusion with other functional polypeptides that could promote and stabilize nanoparticle fabrication. The genetic conjugate of GWH1 and PaDBS1R1, antimicrobial peptide drugs, to GFP-H6 resulted in the formation of 30 nm protein nanoparticles [115]. The engineered GFP nanoparticles displayed a mild antimicrobial effect, which was markedly enhanced by the chemical conjugation of oligo-FdU pentamers to protein nanoparticles. Another study revealed that the presentation of a ligo-Ara-C prodrug, a pentameric form of Ara-C with multivalent engineered GFP-nanoparticles, T22-GFP-H6, enhanced payload delivery to target cells [116]. Recently, a targeted delivery system for Auristatin E was developed by nanoconjugation of Monomethyl Auristatin E (MMAE) with *E. coli* produced CXCR4 targeted T22-GFP-H6 nanoparticles [117]. This novel nanoconjugate demonstrated efficacy against aggressive acute myeloid leukemia in mouse models.

Owing to the potential applications of GFP nanoparticles, the molecular mechanism of this assembly has been revealed for the rational development of new constructs with improved functions. López-Laguna et al. recently studied the architectural role of divalent cations in the assembly of GFP fusion protein nanoparticles (Figure 8A,B) [114]. The assembly and disassembly of protein nanoparticles can be achieved at the nanoscale by controlling the coordination of divalent cations with histidine-rich fusion proteins under ambient conditions [112]. Owing to this stoichiometric manipulation, specific proteins can interact with each other at the molecular level, which enables effective nano-biochemical manipulation of the architecture of the material. Accordingly, the developed polymerization protocol was further extended to bio-fabricate other protein nanoparticles, such as T22-STM-H6 (STM; human Stefin A triple mutant), T22-5CTP-H6 (5CTP; five repeats of the β-chain of human chorionic gonadotropin (hCG))-derived peptide, and T22-HSNBT-H6 (HSNBT; human nidogen-derived protein) [111,118]. Additionally, the conjugation of small drugs, such as MMAE, to these protein nanoparticles resulted in targeted drug delivery abilities.

## 4. Conclusions and Outlook

We reviewed recent studies on the development of novel genetically engineered protein nanoparticles and their utilization as smart drug nanocarriers in the field of cancer therapy. Notably, significant advancements in this field have been made over the past few decades. Cancer and other serious diseases are expected to be significantly affected by the development of therapeutic nanoparticle delivery technologies. Therefore, nanoparticle materials that can safely and effectively deliver drug agents to the target site must be discovered.

The bioengineering of protein nanoparticles offers many opportunities for meaningful progress in the near future. In contrast to chemically synthesized nanocarriers, genetically engineered nanocarriers enable the precise control of chain length and monodispersity and potential alteration of their structure and biosynthesis at the genetic level to achieve precise results. Several protein nanoparticles from natural sources are attractive building blocks for the development of nanocarrier systems. Tailored functionalization of these protein nanoparticles as drug nanocarriers can be employed for their assembly into distinct nanostructure morphologies. Self-assembled protein nanocages can be further modified with multiple functional groups, such as attachment sites, imaging agents, or targeting moieties at the gene level, and produced as recombinant protein fusion nanoparticles for use in vaccine design or drug delivery. Several distinct mechanisms of drug loading and specific targeting have been demonstrated by engineered nanoparticles for the treatment of tumors in vivo.

Interestingly, protein design is becoming more accessible owing to computational advances. Currently, highly homogenous self-assembled nanoparticles can be designed using computational approaches, which could provide new options for vaccine design and drug delivery. However, considerable effort and expertise are still required to repurpose naturally existing nanocages and design novel protein nanostructures.

In addition to the immunogenicity that threatens to induce drug neutralization or rapid clearance of nanotherapeutics, protein nanoparticles are associated with a significant challenge and limitation in the design, manufacture, and scale-up of production. Advances in molecular dynamics simulation and molecular biology techniques could act as master keys to offer new opportunities for nanotherapeutic delivery, which could meet the demands of clinical production efficiency. Therefore, the next era of effort and research is expected to result in the development of protein nanoparticles, natural sources, or de novo design through genetic engineering steps to establish better therapeutic carriers.

## Data Availability

Not applicable.
